# Neural Mechanisms of Time-Based Prospective Memory: Evidence for Transient Monitoring

**DOI:** 10.1371/journal.pone.0092123

**Published:** 2014-03-18

**Authors:** Kevin M. Oksanen, Emily R. Waldum, Mark A. McDaniel, Todd S. Braver

**Affiliations:** Washington University in St. Louis, St. Louis, Missouri, United States of America; University College London, United Kingdom

## Abstract

In daily life, we often need to remember to perform an action after, or at, a specific period of time (e.g., take pizza out of oven in 15 minutes). Surprisingly, little is known about the neural mechanisms that support this form of memory, termed time-based prospective memory (PM). Here we pioneer an fMRI paradigm that enables examination of both sustained and transient processes engaged during time-based PM. Participants were scanned while performing a demanding on-going task (n-back working memory), with and without an additional time-based PM demand. During the PM condition participants could access a hidden clock with a specific button-press response, while in the control condition, pseudo-clocks randomly appeared and were removed via the same response. Analyses tested for sustained activation associated with the PM condition, and also transient activation associated with clock-checks and the PM target response. Contrary to prior findings with event-based PM (i.e., remembering to perform a future action when a specific event occurs), no sustained PM-related activity was observed in anterior prefrontal cortex (aPFC) or elsewhere in the brain; instead, transient clock-related activity was observed in this region. Critically, the activation was anticipatory, increasing before clock-check responses. Anticipatory activity prior to the PM target response was weaker in aPFC, but strong in pre-Supplementary Motor Area (pre-SMA; relative to clock-check responses), suggesting a functional double dissociation related to volitional decision-making. Together, the results suggest that aPFC-activity dynamics during time-based PM reflect a distinct transient monitoring process, enabling integration of the PM intention with current temporal information to facilitate scheduling of upcoming PM-related actions.

## Introduction

Haley put frozen pizza in the oven to cook, but because he oven timer was broken she needed to keep track of the time herself. She then went about her house cleaning, but every so often checked a clock. Approximately fifteen minutes later she remembered to take the pizza out of the oven before it burned.

This everyday example illustrates the effective use of a particular prospective memory (PM) function, termed time-based PM, which is omnipresent in our daily lives. Time-based PM involves remembering to perform an action after a specified amount of time has elapsed, or at a particular time of day (e.g. remembering to call a friend at 2:00 p.m.) [1], [2]. Haley, in the case above, exhibits successful time-based PM when she remembers to remove her pizza from the oven after it had baked for fifteen minutes. Just as Haley successfully performed her time-based PM task despite attending primarily to her house-cleaning, most PM tasks performed in daily life are interleaved with numerous attention-demanding tasks. As such, time-based PM tasks are studied under similar attentionally-demanding conditions in the laboratory. Namely, participants are asked to make a target response after a set time, while also performing a cognitively demanding ongoing task activity (e.g., answering trivia questions, performing an n-back working memory task, or doing pattern discrimination tasks [2]3,4]. Moreover, paralleling our everyday example, participants are allowed to initiate clock checks (the clock is not continuously displayed) during the time interval.

Most of the research on time-based PM has focused primarily on behavioral outcomes; very little work involving neuroscience methods, such as ERP [3], [4] or PET/fMRI [5] has been conducted. This represents a significant limitation, given that brain-based approaches can provide powerful insights into the possibly unique mechanisms that underlie time-based PM. In the present study, we utilize fMRI to inform theoretical hypotheses regarding the neural and cognitive processes that support time-based PM, and how they might be similar to or different from other forms of PM.

In particular, there is a larger body of literature, both behavioral and neuroscience-based, that has focused on event-based prospective memory (event-based PM). Event-based PM tasks require that participants perform an action in response to an external cue (e.g., remembering to give a co-worker a message when you see her). An influential theoretical framework of event-based PM has been the multi-process account, which postulates that the nature of the external cue and its relation to the task demands drive the processes by which event-based PM tasks are performed [6]. For example, in some experimental event-based PM paradigms, the PM cue is not directly related to on-going task processing, such as when the PM cue is a particular syllable (e.g, “tor”) and the on-going task requires semantic processing of presented words. In these event-based PM paradigms, which have been termed *nonfocal*, sustained attentional monitoring for target PM events appears to be required. In behavioral studies, the evidence for such sustained monitoring processes is the presence of a performance cost on the ongoing activity; that is, responses to the on-going task (on non-PM trials) are made more slowly in the presence of PM demands, compared to when the PM demands are absent [7], [8]; [9]–[11]. Converging evidence for sustained monitoring comes from neuroimaging studies, which have reported sustained activity in the anterior prefrontal cortex (aPFC), dorsolateral PFC, and other components of the dorsal frontoparietal attentional control system during nonfocal event-based PM tasks compared to control tasks [12]–[14].

In contrast to nonfocal event-based PM, wherein PM cue detection requires processing that is irrelevant to the ongoing task, in *focal* event-based PM paradigms, processing of cues occurs naturally as part of the ongoing task. For example, detecting a specific word (e.g., “tornado”) during a lexical decision task is considered a focal task because word reading is primary for both the ongoing lexical decision task and PM cue detection. The theoretical premise is that the critical features of a focal PM cue are processed during the normal course of performing the ongoing activity, thereby stimulating relatively spontaneous retrieval of the associated PM intention [15]; (see also [16], for theoretical specifics of spontaneous retrieval). In short, successful focal PM performance is thought to involve spontaneous retrieval processes (triggered by the appearance of the PM event), rather than sustained attentional monitoring. Indeed, as predicted by the multiprocess account, in some focal event-based PM studies, performance costs to the ongoing task when a PM task is present are minimal or absent [8], [17], [11]. However, these findings are not decisive, as the cost index may lack sensitivity to monitoring, or indeed may reflect processes other than monitoring (see [18], [19]).

Stronger empirical support for the assumption that spontaneous retrieval supports focal PM performance was provided in a recent fMRI neuroimaging study that contrasted matched focal and nonfocal event-based PM conditions [12]. In this study, focal event-based PM was associated with an absence of sustained activation anywhere in the brain; instead a strong pattern of transient activity was observed during successful PM events. This transient activity occurred not just in components of the dorsal frontoparietal network, but also in a ventral brain network, sometimes termed the salience network, associated with detection of salient events and a bottom-up shift of attention [20], [21]. As such, the fMRI results suggest that sustained attentional monitoring and spontaneous retrieval can be clearly distinguished in terms of their contrasting neural signatures. Thus, the prior literature supports a multi-process account of event-based PM in which sustained top-down attentional control is required for nonfocal event-based PM performance whereas focal event-based PM tasks can be supported by spontaneous retrieval processes (see [22] for a review). However, it remains unclear whether time-based PM tasks can be aligned with this distinction, or instead engages distinct neurocognitive mechanisms.

One theoretical possibility is that the top-down, self-initiated processing required by time-based PM for intention maintenance and monitoring of time, including decisions to initiate clock checks, poses an even greater reliance on sustained attentional processes than does event-based PM [23]. According to this account, participants perform time-based PM by continually monitoring the passage of time (both internally and with external timing devices). If indeed time based PM does rely on sustained attentional processes, in our original example, Haley’s success in removing the pizza from the oven on time depended on her maintaining the intention in mind (assuming she did not encounter environmental cues that reminded her of the pizza; see [24]), monitoring the passage of time, and initiating clock checks. For current purposes, the expectation is that performance of time-based PM tasks should be associated with a neural signature of sustained monitoring similar to that observed in nonfocal, event-based PM: a robust pattern of sustained activity within aPFC and other components of the dorsal frontoparietal attention network

In terms of potential support for this theoretical interpretation, the prior literature on time-based PM is somewhat sparse and mixed. With regard to the presence of sustained monitoring in time-based PM tasks, some time-based PM studies have found PM performance costs (e.g., [25], [26]) consistent with sustained monitoring, but other studies have reported no costs (e.g., [27], [5]). Given the very small number of studies that have investigated costs in time-based PM, and the wide variability of task conditions used in these studies, the available reaction time findings do not clarify whether sustained monitoring is required for the performance of time-based PM tasks. Moreover, there has been very limited neuroscience-based research on time-based PM [3]–[5]. The work that has been conducted used methods such as ERP and PET that preclude targeting and isolation of sustained monitoring processes. Although ERP methods provide enhanced temporal resolution, they lack spatial specificity and are ineffective in detecting block-wide sustained activity. Conversely, PET provides greater spatial specificity, at the cost of temporal resolution, thus proving to be ineffective in detecting transient activation. Accordingly, it remains uncertain to what extent sustained PFC activity is associated with time-based PM in a typical circumstance when an external clock is available for checking, as in the current paradigm.

In contrast to the sustained monitoring prediction outlined above, it is also possible that PM performance in time-based PM engages neurocognitive mechanisms that are somewhat distinct from those present during event-based PM. Specifically, time-based PM tasks appear to involve a volitional component involving clock-check decisions. Such volitional processes might best be described as transient monitoring, as they fall somewhere between the sustained monitoring sometimes observed with nonfocal event-based PM and the absence of monitoring often associated with focal event-based PM. In particular, during time-based PM, transient monitoring processes might be engaged during the retention interval, a pattern not seen in event-based PM, at least in fMRI studies [13]. This form of transient monitoring contrasts with the transient neural activations reported in focal event-based PM, as these latter activation patterns are reactive; that is, tied to the appearance of focal PM cues [12]. Instead, we hypothesize that in time-based PM the transient activity related to clock-checking is self-initiated (i.e., volitional), and thus may be characterized by a distinct neural signature.

This transient monitoring account of time-based PM is consistent with the test-wait-test-exit (TWTE) description of time-based PM proposed by Harris and Wilkins [28], and originally developed more generally by Miller, Galanter and Pribram [29]. The idea is that, because sustained monitoring exacts significant attentional costs, an individual would only periodically monitor (test) to evaluate whether the moment is appropriate for performing the intended action. If a test revealed that it is too early to perform the action, then the individual would return full attention to the ongoing task (wait) and some time later initiate another test.

Although overt clocking checking behaviors are consistent with the TWTE account [8], [28], the available behavioral results leave uncertain whether sustained monitoring processes prompt periodic clock checks or whether the underlying processes that initiate a clock check are transient. Neuroimaging work on volitional decision-making, in which participants are asked to periodically make a free choice to engage in a particular task or action, has revealed transient, anticipatory activity occurring prior to the choice selection in a frontoparietal network, that also includes aPFC, along with the pre-SMA [30]. If similar volitional and anticipatory processes occur during time-based PM, we would not predict sustained activation throughout the task, but instead should observe transient activation in aPFC, pre-SMA, and other components of the dorsal frontoparietal monitoring network that occurs reliably in advance of clock-check actions.

In the present study we were additionally interested in investigating neural activity related to execution of the PM target response (i.e., performance of the intended action). We thought it possible that further dissociations between time-based PM and event-based PM might emerge here. Specifically, in event-based PM tasks, target responses are required when a particular PM cue is present. By contrast, in time-based PM tasks, there is no explicitly presented cue for performance. Instead, participants must not only self-initiate clock-checks, but they must also self-initiate PM target responses. Interestingly, in our prior event-based PM study, although we observed sustained aPFC activity selectively in the nonfocal condition, we also observed transient aPFC activity related to the PM target response in both the focal and nonfocal conditions [12]. One interpretation of this PM-target-related transient activity is that it reflects the rapid activation and initiation of a PM-target response that is triggered (i.e., released) by the PM-cue. However, in time-based PM, it may be the case that the PM-target response does not need to be triggered in the same way, since it can be more gradually pre-activated by transient monitoring processes (i.e., clock-check events) that are proximal to the PM-target period. Under this account, we would predict that time-based PM would further dissociate from event-based PM, with aPFC showing weak, rather than strong transient PM-target related activation (as compared to transient clock-related activity).

The present study was designed to test these theoretical hypotheses regarding neurocognitive mechanisms of time-based PM. In particular, we contrasted sustained (i.e., block-related) brain activity while participants performed a time-based PM task in the context of an on-going task relative to a matched control condition (on-going task alone). Further, we examined transient activation both prior to and following self-initiated clock checks in the time-based PM condition relative to similar clock events in the control condition. We also examined transient activation associated with PM target responses. The sustained monitoring account predicts sustained activity during time-based PM in a network of regions similar to that found selectively for nonfocal event-based PM [12], [13], [31].The transient monitoring account predicts that these regions should be engaged during time-based PM but only in a transient manner, and primarily in anticipation of and during self-initiated clock-checks. Finally, we expect to observe a further distinction with event-based PM in terms of activation associated with execution of the PM target response. Specifically, because time-based PM task performance must be self-initiated, we predicted a weaker response to PM-target events themselves (possibly due to pre-activation by prior clock-checks) relative to the activation associated with the externally cued event-based PM target events.

## Methods

### Ethics

Written consent, as well as demographic information, was obtained from participants prior to their participation in the study. These files were stored in a locked filing cabinet, within a locked office. Participation was also tracked using a password-protected document, on a password-protected computer, within a locked office. The consent process and study protocol was extensively reviewed, and approved by the Washington University in St. Louis School of Medicine Human Research Protection Office prior to the beginning of data collection (Approval number 201106419).

### Participants

Twenty-four young adults were recruited from Washington University, and the surrounding community (11 Males, 13 Females ranging in age from 19–36, M = 24). Participants were included if they were right-handed, native English speakers, had normal visual acuity (potentially corrected with glasses or contacts), had no prior history of neurological or psychological disorders or history of illicit drug use, were not currently taking psychotropic medication, and had no medical conditions contraindicated for fMRI scanning. Participants were compensated $25/hour for the fMRI scanning sessions. Three participants were excluded from analysis, 2 for not completing the scanning session, one for excessive movement. (2 Females, 1 Male) An additional participant showed poor task performance (2.6 standard deviations below the mean), thus may have represented an outlier. Consequently, behavioral analyses are reported with this participant excluded. The imaging analyses were conducted both with and without exclusion, with no difference in results; to be conservative, primary statistical effects are reported with the participant excluded.

### Task

The experiment was programmed in Eprime (version 2.0.8), and consisted of two conditions, control and time-based PM. In both conditions, participants performed the 2-back version of the N-back working memory paradigm as the on-going task [32]. In this task, participants were asked to determine whether the current visually presented word (presented in lowercase for 1.5s) matched the word presented two trials back. They were told to press a button with the index finger of their right hand when the currently presented word matched the word presented two trials back (target item), and another button with their right middle finger when the presented word was not a 2-back match (non-target item). The stimuli consisted of 616 words presented in black, Arial bold font, size 32 (e.g., **BAND**). Each of the 616 words was placed onto one of 16 lists. Eight of these lists were comprised of 131 words (associated with the 3-minute block), and the remaining eight lists included 171 words each (associated with the 4-minute block). Further, half of each of the list types (4 each) were used for the control condition, and half for the time-based PM condition, with list assignment counterbalanced across participants. In each list, 30% of the items were targets (2-back matches) and 70% were non-targets. Further, 14% of the non-targets were lures, meaning that the word presented matched a previous recently presented word, but not the word that was presented 2 trials back.

**Figure 1:**
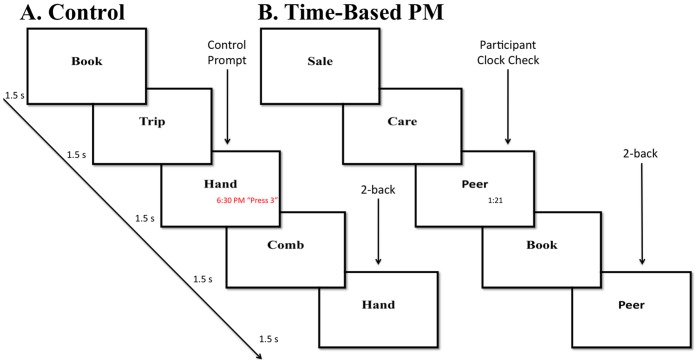
Task Design. Participants performed the 2-back condition of the n-back working memory task in either a control condition (Panel A) or time-based PM condition (Panel B), in counterbalanced order. The 2-back task presented a continuous sequence of words at a regular rate (1.5s) with target responses required when the current item matched that presented 2 trials back (arrow labeled “2-back”). In the control condition, at intermittent times, a non-informative clock randomly appeared (red font) with the instruction “press 3” (indicating the relevant button to press on the response box). Upon reacting to this prompt the clock would disappear. (arrow labeled “Control Prompt”). In the time-based PM condition, participants self-initiated a clock-check with the same button-press response (arrow labeled “participant clock-check”), leading to the appearance of an informative clock indicating the elapsed time since the start of the task block. They were asked to use this information to make a target response after 3 or 4 minutes. The 2-back task continued for an additional 20 seconds following the designated target time.

The PM and control conditions were performed in either 3-minute or 4-minute task blocks. In the time-based PM condition, participants performed the 2-back task, but were additionally given the ability to check an ongoing clock by pressing a third button with their ring finger, and the PM task of pressing a fourth button (with the pinky finger of their right hand) when a specific temporal interval (either exactly 3 minutes or exactly 4 minutes) had elapsed (see Figure 1). Note that the actual task block durations were longer than the PM target durations (3.33 minutes and 4.33 minutes, respectively). Thus, participants were required to continue the n-back task for an additional 20 seconds following the target PM response time. This also enabled the detection of late or missed PM target responses.

Clock events occurred in both the control and time-based PM blocks, however the control of these clock events and the temporal information presented during the clock events differed across the two conditions. Specifically, during time-based PM tasks, a self-initiated key press (button three with the ring finger) led to the visual presentation of a clock denoting the elapsed time since block onset (e.g., 1:20 min). The clock information appeared in red Arial font, size 20 below and to the right of the presented n-back item. In the control condition, pseudo-clock information was presented intermittently and in the same screen location, but here the clock information was irrelevant to the task and consisted of random times [e.g. 6:30 PM]. Moreover, rather than appearing in response to the 3-button press, as in the time-based PM condition, in the control condition the clock appeared automatically at pseudo-random intervals along with the instruction “Press 3”. This instruction appeared with every control clock appearance. Thus, the control condition did not involve PM, as the participant was not required to remember the relevant clock response. The timing of pseudo-clock appearances in the control condition was determined by the observed timing and frequency of clock-checking during a time-based PM pilot study. Thus, the clock presentations in the control condition approximated typical time-based PM clock-checking behavior.

### Procedure

Prior to entering the scanner, participants first received instructions and practice with the n-back task (using a 50 item list). Upon completion of the initial practice session, participants received additional instructions for the control and time-based PM tasks, and were familiarized with the clock responses in each. They then received additional practice in each condition (again with two 50-item lists: one each for the control and time-based PM tasks)

In the scanner, participants completed four time-based PM and four control scanning runs. Each scanning run consisted of two task blocks, one of 3-minute and the other of 4-minute duration, with the order of the two durations counterbalanced across runs and participants. Each task block began with an instruction screen (presented for 7.5 seconds to allow ample time for encoding), that indicated the task condition, and for the time-based PM condition, the time to execute the PM intention (either 3 minutes or 4 minutes),. Finally, in each scanning run, a resting fixation block (30 seconds duration) occurred before and after each task block.

The scanning session took place in a 3T Siemens TRIO full body scanner at Washington University’s Center for Clinical Imaging Research. A pillow and foam pads were used to minimize movement in the twelve-channel head coil. Participants additionally wore sound dampening headphones, which enabled communication. Both BOLD (Blood-oxygen Level Dependant) and anatomical images were acquired for all participants. High-resolution anatomical images were acquired with a MP-RAGE T1 weighted scan [repetition time (TR) 2.4s, echo time (TE) 3.16ms, flip angle (FA) 8, 256 x 256 acquisition matrix,176 slices, voxel size 1x1x1 mm]. Functional BOLD images were acquired using an interleaved gradient echo-planar imaging sequence [TR 2.5s, TE 25ms, FA 90, 256 x 256 acquisition matrix, 225 volumes, 34 slices, voxel size 4x4x4mm] with slices acquired in an oblique axial orientation aligned with the AC-PC plane, to, allow full-brain coverage with high signal to noise ratio.

### Data Analysis

#### Behavioral

Behavioral data were extracted from the Eprime files and analyzed using Microsoft Excel and SPSS. Time-based PM accuracy can be operationalized in a variety of ways. Previous investigators have suggested examination of accuracy in terms of whether a time-based PM response occurred within a specified window around the target time, using both liberal and more stringent criteria [33]. Accordingly, we examined performance in terms of both a 6-second (+/– 3 sec) and 20-second (+/– 10 sec) window around the target time. Additionally, we examined clock-check frequencies in the time-based PM condition, as a function of time-to-target, in 1-minute bins, and in relation to PM accuracy and fMRI activation.

N-back task accuracy and reaction time were analyzed and compared across the time-based PM and control conditions. Additionally, n-back performance in the time-based PM and control conditions was compared during pre-clock epochs [7.5 secs (5 n-back trials prior to the clock-check)] to determine how on-going task performance was impacted by endogenous preparation of a clock-check. The 7.5 sec window length was selected based on an initial examination of the fMRI data, which suggested increased activity in the time-based PM condition compared to the control condition during this pre-clock period (see Results). We excluded all n-back trials occurring during clock-checks from all the performance analyses because we were primarily interested in how endogenous cognitive processes (rather than the physical clock-check action) influenced n-back performance.

#### fMRI

All fMRI data were analyzed using in-house software. Image preprocessing occurred through the Central Neuroimaging Data Archive [(CNDA) Neuroinformatics Research Group 2004] pipeline, and included slice-time correction, motion correction through realignment to the first image, co-registration of participants’ mean image to their own structural T1, spatial normalization to a standard atlas space, and smoothing using a 9mm FWHM Gaussian filter. Note that the pre-processing procedure excluded a high-pass filtering step, as such filtering might attenuate sustained activity.

A generalized linear model (GLM) approach was used to estimate simultaneous and independent parameter values for sustained (block effects) and transient (event related) effects [34]. Separate block regressors coded for the time-based PM and control blocks, relative to the fixation block baseline, modeled as a boxcar convolved with a standard hemodynamic response function (HRF). The primary transient events of interest were the free response clock-checks occurring in the time-based PM condition (as well as the comparable clock responses in the control condition). The behavioral results indicated the typical time-based PM pattern of highly increased clock-checking frequency occurring in the last minute proximal to the time-based PM target time. Given that the frequent clock-checks preceeding the target time could represent a distinct psychological process from that associated with checks that occur earlier in the block, we chose to take a conservative approach and focus our analysis on the early block checks. Thus, the clock-checks (and the control clock presentations) occurring in the final minute prior to the target time were modeled with a separate regressor. Likewise, to test whether time-based PM clock-check events might reflect a transient monitoring process, we were interested in examining time-points prior to, as well as following, a clock-check event. Consequently, we choose to model clock events (both clock-checks in the time-based PM condition and clock presentations in the control condition) using a peri-event unassumed response shape, in which the clock onset was centered in an epoch of 35 sec (i.e., 14 TRs, with the clock-onset occurring at the start of TR8, modeled with a set of 14 FIR basis set regressors). PM target responses were also modeled in a similar fashion, so that they could be directly compared to clock-check events. Finally, we modeled the instruction cue period as a single event (with duration 7.5s), convolved with a HRF.

After computing the GLM for each subject, group level random effects analyses were conducted to identify sustained and transient activation. Our primary hypothesis was that the monitoring processes engaged during time-based PM would involve the same aPFC regions that have been most consistently associated with monitoring during event-based PM. Thus, our first analyses were ROI-based and used a seed within lateral anterior prefrontal cortex (aPFC). Prior meta-analysis has identified this aPFC seed (centered on coordinates: x = +/– 34 y = 56, z = 9) as a location consistently engaged by PM tasks, episodic memory retrieval, working memory, and multitasking [35]. The ROI was created with a 8mm-radius sphere centered on these coordinates, after masking out non-brain voxels.

Sustained monitoring effects were examined to determine whether aPFC exhibited block-related activity, and further, whether a time-based PM > control pattern was present. Transient monitoring effects were tested by examining aPFC activation during the clock-checking period for a time-based PM > control pattern (in terms of a significant condition x time interaction). In addition, we were interested in the time course of activation to determine whether time-based PM-related activation was also present in aPFC prior to clock-checking, consistent with a transient monitoring process. Finally, we were interested in the pattern of aPFC activation in relation to PM-target responses. We tested this by comparing aPFC activity dynamics during the PM-target response period with the clock-checking period (again in terms of the condition x time interaction).

In addition to the ROI-based analyses, a whole-brain exploratory analysis was also conducted, with appropriate FWE-correction for multiple comparisons, using a Monte Carlo simulation approach to define appropriate statistical thresholds and minimum cluster size criteria. The specific contrasts and thresholds used for these analyses are described below under Results.

## Results

### Behavioral

**Figure 2:**
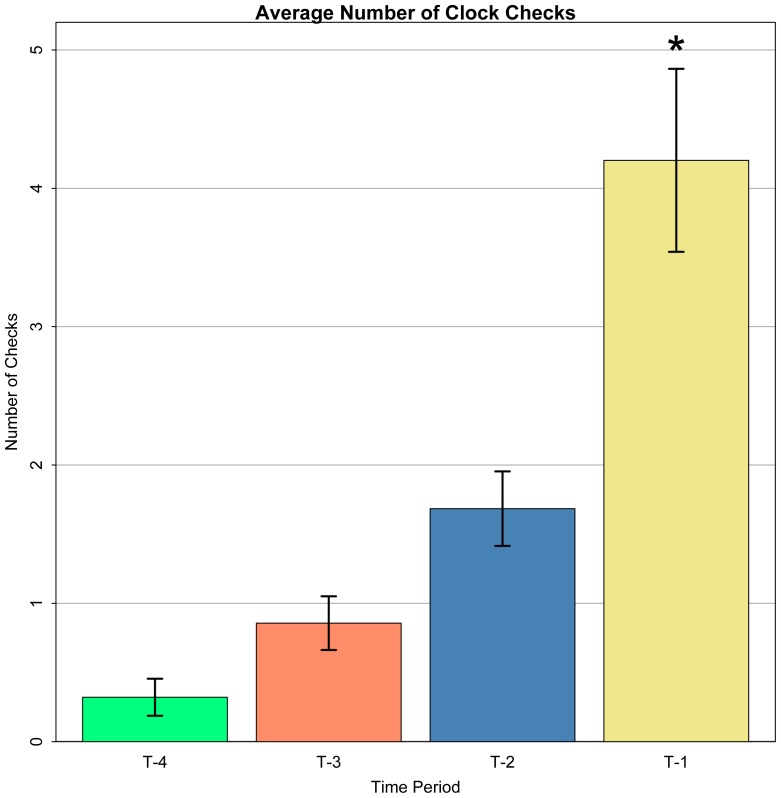
Clock-check responses across time . The mean number of clock-checks in each minute during TBPM condition is displayed (referenced to the target time; e.g., Target-1 refers to last minute prior to target time). Clock-checks reliably increased in frequency with increasing proximity to the PM target time. Clock-checks in the final minute (Target-1) were significantly greater than all preceding minutes combined, consistent with prior findings, indicated by a * over the T-1 period (p<.05). ^+^ Target-4 only contains data from 4-minute trials. 3-minute trials were shifted accordingly to align the last minutes.

Participants were able to successfully integrate time-based PM demands with on-going task performance. With a liberal criterion (+/– 10 secs from the target time), PM accuracy was relatively high (M = .88, SD = .15), and remained so even with applying stricter criteria ( +/– 3 sec window; accuracy  = .76, SD = .24). Participants exhibited intermittent clock-checking behavior prior to the target time (total number of clock checks: M = 54.67, SD = 40.69, range: 12 – 162]. Consistent with previous studies, clock-checking frequency increased (Figure 2) as the time-based PM target time approached (Einstein et al 1995). In fact, participants checked the clock more often in the final minute prior to the target time (M =  4.23, SD = 3.11) than in all the minutes prior to that combined (M = 2.73, SD = 2.42), t(19)  = 3.07, p = .006. There was no significant difference in the number of participant clock checks, compared to the number of control presses (t(19) = 1.08). Clock checking behavior was examined in relation to target accuracy as well as fMRI activation. The overall trend of clock checks in relation to target accuracy was in the expected direction (in that more frequent clock checking was associated with greater target accuracy); however, the correlations were not statistically significant (r = .102). Clock-checking frequency was also not reliably related to fMRI activity, in any of the analyses reported below. However, it is possible that the null effects were related to low power in our study for detecting correlations [36], [37]. Performance on the time-based PM task did not come at the cost of performance on the on-going 2-back task. In the time-based PM task, 2-back accuracy was high (M = .90, SD = .04) and reaction time was relatively quick (M = 564, SD = 77). The performance levels were nearly identical to those in the control condition, for both accuracy (M = .90, SD = .05; t(19)<1) and RT (M = 558, SD = 81; t(19) <1). These data are inconsistent with the hypothesis that time-based PM involves sustained monitoring processes, as sustained monitoring should incur a performance cost during the PM task blocks.

To examine whether performance results may instead point to the possibility that transient monitoring may lead to subsequent clock-checking behavior, we examined the 5-trial window immediately prior to clock events in the time-based PM and control conditions. This analysis revealed no difference in RT across conditions during the five trials preceding clock events (time-based PM: M = 571, SD =  87; Control: M = 580, SD = 78; t <1). However, surprisingly, n-back performance was more accurate prior to clock-checks in the time-based PM condition (M = .92, SD = .05), compared to clock events in the control condition (M = .89, SD = .06; t(19)  = 2.25, p = .036). Although the direction of the effect suggests a time-based PM performance benefit, rather than a cost, it is consistent with the hypothesis that transient changes in processing occur prior to clock-onset in the time-based PM condition. To be consistent with the fMRI data analysis, the performance analyses reported here exclude both n-back trials performed in the final minute prior to the target time and those performed after the target time. However, it should be noted that the pattern of results is virtually unchanged if these trials are included.

### Sustained fMRI Activity

We first tested whether aPFC showed block-related activity consistent with a pattern of sustained monitoring. Block-related estimates from the aPFC ROIs were submitted to a 2 region (right aPFC, left aPFC) x 2 condition (time-based PM, control) ANOVA. Neither the main effect of condition (F = 1.83 p = .192) nor the region x condition interaction (F = 3.60, p = .073) were significant. However, there was a main effect of region, due to greater block-related activity in the right aPFC (F = 5.461, p<.05). The right aPFC did show weak block-related activation associated with the on-going n-back task (t = 1.5 p = .1469), but no hint of difference between time-based PM and control conditions. Thus, there is no evidence based on aPFC activation for a sustained monitoring account.

**Figure 3:**
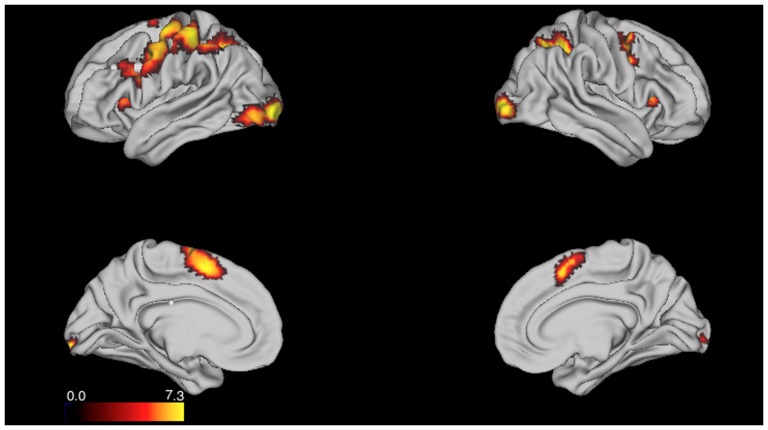
Block-related activation associated with on-going 2-back task. Anatomical location of regions showing significant block-related activity in both Control and time-based PM conditions identified through a conjunction analysis. Color scale indicates minimum z-value across the two conditions. Prominent foci of activity are seen in lateral and medial prefrontal and parietal cortex, consistent with prior n-back findings.

To more comprehensively test this interpretation, we conducted a whole-brain contrast, with appropriate multiple comparisons correction (voxel-wise threshold of z = 3.0 and a minimum cluster-size of 42 voxels, providing image-wise false-positive correction at p<.05). From this threshold, no regions showing a time-based PM > control pattern (or the reverse) were identified. Importantly, this is not to say that block-related activation was not present at all during this task, just that it did not differentiate between the two conditions. Indeed, when testing for regions showing significant activation during both time-based PM and control blocks (i.e., a conjunction analysis), we observed the standard activation of the dorsal frontoparietal control network typically observed during n-back tasks [38], including lateral and medial prefrontal and parietal cortex (Figure 3). Thus, the whole-brain analysis supports the results of the ROI analysis in indicating that block-related activity patterns are inconsistent with sustained monitoring occurring during time-based PM.

### Transient Clock-Related fMRI Activation

**Figure 4:**
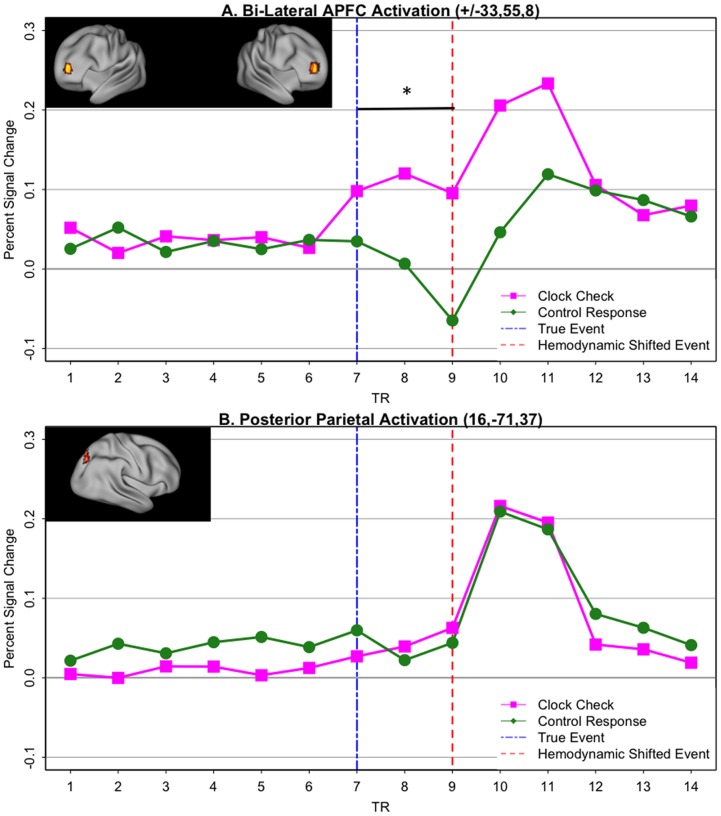
Peri-event timecourses of clock-related activation. Timecourses refer to 14-TR epochs (35 seconds) centered at the actual event onset time (TR-7, Blue Dashed Vertical Bar) A. Bilateral aPFC ROI (anatomical location shown in inset), exhibits a reliable increase in activation for time-based PM clock-checks (Purple) relative to Control clock-activation (Green). Two peaks of activation are present, one preceding clock onset (vertical Red dashed line at TR-9, adjusted to indicate hemodynamic response lag) and the other following it. The horizontal bar indicates that a significant PM > Control pattern was observed during the anticipatory, pre-clock period (p<.05). B. Control region in posterior parietal cortex (anatomical location shown in inset) that showed no difference in post-clock activation between time-based PM and Control conditions, as well as no pre-clock activation in either condition.

We next tested whether aPFC showed clock-related activity consistent with a pattern of transient monitoring. As described in the Methods, we extracted peri-event clock-related activity (i.e., preceding and following clock onset) without making assumptions regarding the hemodynamic response function (i.e., using an FIR basis set approach) as a 14-timepoint epoch (with the clock onset occurring at the beginning of timepoint-8). Clock-related timepoint estimates from the aPFC ROIs were submitted to a 2 hemisphere (right aPFC, left aPFC) x 2 condition (time-based PM, control) x 14 timepoint ANOVA. There was both a significant main effect of time (F = 4.25, p<.001), as well as a condition x time interaction (F = 4.68 p<.001). No main effect or interactions of hemisphere were observed (all F’s<1). As depicted in Figure 4a (which collapses across hemisphere), the main effect of time emerged as a result of, increased post-clock activity occurring in both time-based PM and control conditions. The time x condition interaction indicates greater activation in time-based PM condition compared to the control condition. Moreover, in the time-based PM condition there appeared to be two distinct peaks of activity, the first at timepoints 7,8,9 and the second at timepoints 10,11. Importantly, the second peak of activation is consistent with a post-clock onset response, which also is apparent in the control condition, taking into account the 5-7.5 second (2 timepoint) lag in the hemodynamic response. The first peak, which selectively occurred in the time-based PM condition, appears to be consistent with pre-clock activation related to transient monitoring and the endogenous preparation of a clock-check response. Supporting this idea, a second analysis restricted to this putative pre-clock related activation (i.e., timepoints 7-9) produced a main effect of condition (time-based PM > control; F = 5.6, p<.05).

We next conducted a whole-brain voxelwise analysis to identify whether other regions in the brain showed similar clock-related activation to that observed in aPFC. For this analysis, we identified regions via the condition x time interaction analysis (again multiple comparisons corrected with voxel-wise z = 3 and minimum cluster size of 42 voxels, providing image-wise false-positive protection at p<.05). To further restrict the findings to regions showing evidence of pre-clock related activity, we applied an additional conjunction contrast of time-based PM > control at timepoints 7–9 (here using an uncorrected, p<.05 and 10 voxel threshold, for additional sensitivity), based on the aPFC findings suggesting an early activation peak during this period. This analysis identified 7 regions showing the predicted pattern (see Table 1 and 2). Of these, two regions were bilateral aPFC, confirming the original ROI-based analyses (Table 1). The other regions included bilateral dorsolateral PFC, pre-supplementary motor area (pre-SMA), and superior parietal cortex (Table 2). Thus, the other regions that showed the same pre-clock time-based PM-related activation were all part of the same dorsal frontoparietal network consistently engaged by attentional control and monitoring processes [39].

**Table 1:** Main Effect of Clock Event: TBPM>Control.

Region	X	Y	Z	Voxels	Z-score
Right APFC (BA 10)	34	56	9	30	2.17
Left APFC (BA 10)	–34	56	9	30	2.07

**Table 2:** Main Effect of Clock Event: TBPM>Control.

Region	X	Y	Z	Voxels	Z-score
Right APFC/DLPFC (BA 9/10/46)	33	43	25	291	4.46
Left APFC (BA 10)	–32	50	15	73	3.93
Left DLPFC (BA 8/9)	–38	30	39	10	4.49
Left DLPFC (BA 9)	–43	17	38	12	3.89
Pre-Supplementary Motor Area (BA 6/8)	1	20	50	84	6.97
Medial Superior Parietal Cortex (BA 7)	–3	–65	57	24	6.82
Superior Frontal Cortex (BA 6)	–28	–1	63	26	6.35

It is worth noting that the pattern observed in aPFC and other frontoparietal attention regions was quite selective, rather than a general feature of clock-related brain activation dynamics. Indeed, in most of the regions showing clock-related activity (identified through the main effect of timepoint contrast), there was no initial time-based PM clock-related peak, and in fact, clock-related activation was equivalent in both the time-based PM and Control conditions. An example of this pattern, which was present throughout visual, motor, parietal and posterior frontal regions, is shown in Figure 4b.

Finally, the primary analyses excluded clock-checks occurring in the final minute of time-based PM (and also Control) blocks, as a conservative step, in case the frequent clock-checks occurring during this period reflected a qualitatively distinct process. However, when we examined clock-related activity in this last minute for all of the identified regions, we found that the pattern was in fact highly similar for all of them, albeit statistically less robust. This indicates that the primary results were not related to the exclusion of the final minute of clock-checks.

### Transient fMRI PM-response activation

**Figure 5:**
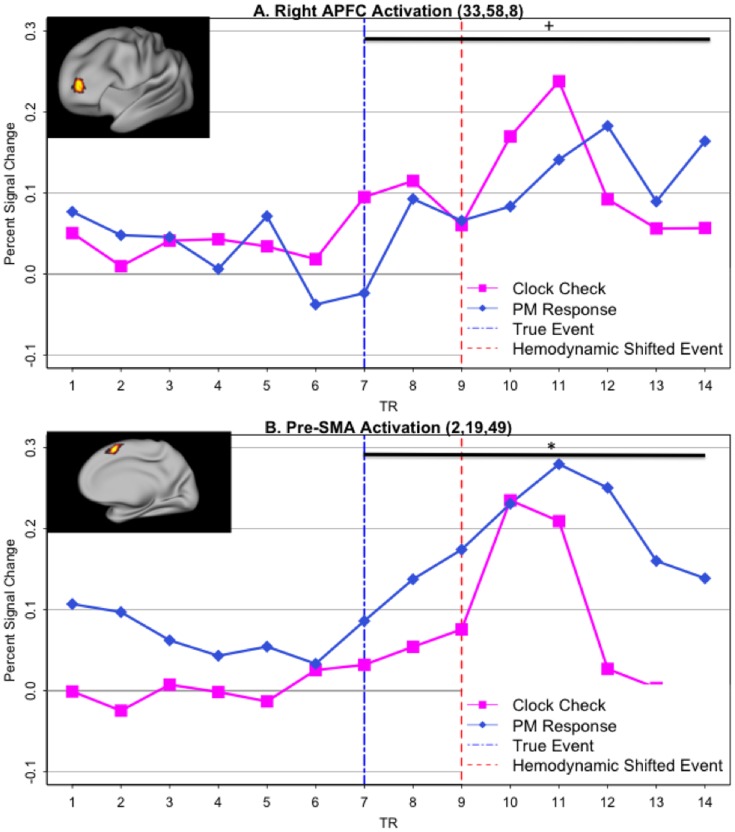
Peri-event timecourses of PM-response activation. Timecourses refer to 14-TR epochs (35 seconds) centered at the time of PM-response/clock-onset (TR-7, blue dashed vertical bar) A. Right aPFC ROI (location shown in inset), exhibiting weaker activation for time-based PM responses (navy) relative to clock-checks (purple). Activity is reduced and delayed both prior to the PM response (TRs 7–9) and following it (TRs 10–14). The horizontal bar indicates the period in which a trend-level significant (p = .07) condition x time interaction was observed. B. Contrasting pattern observed in pre-SMA (location shown in inset), with greater activation for PM responses relative to clock-checks. The horizontal bar indicates the period over which a significant condition x time interaction was observed (p<.05).

We next examined activation occurring in relation to PM-target responses. In contrast with the pattern found for clock-checks, in the aPFC ROIs the transient activation pattern related to PM-target responses was rather weak. Specifically, anticipatory activity (i.e., timepoints 7–9) was reduced relative to what was observed for clock-checking, and the later activation peak appeared delayed and more gradual (i.e., timepoints 10–14) than that observed for the clock-onset response (Figure 5a), This was confirmed by the presence of a marginally significant condition X time interaction when comparing the clock-checks and PM-target response at these timepoints (7–14; F(7,133)  = 1.94; p<.07).

A whole-brain voxelwise analysis (using the main effect of timepoint, and the same multiple comparisons corrected threshold of z = 3, 42 voxel clusters) confirmed the absence of PM-target related activity in the vicinity of aPFC. However, this analysis also suggested that PM-target activity was generally robust in many regions of the brain, including other components of the frontoparietal network engaged by clock-checking. We directly confirmed this via an overlap (conjunction) analysis, to identify regions showing both significant clock-check and PM-target activations. Two regions were identified – right dorsolateral PFC and pre-SMA. Strikingly, these regions actually showed a stronger activation pattern to PM-targets than to clock-checks (timepoints 7–14; condition x time: F(7,133) = 2.21; p<.05; see Figure 5b), contrasting with the pattern observed in aPFC. Indeed, this double dissociation across the 4 ROIs (bilateral aPFC, right dorsolateral PFC, pre-SMA) was confirmed via a region x condition interaction (averaging over timepoints 7–11; F(3,57)  = 2.68; p  = .05).

## Discussion

This study was conducted to investigate the question of whether time-based PM is characterized by the types of sustained monitoring processes that have been observed in nonfocal event-based PM, or instead whether it engages a distinct transient form of monitoring that is more similar to other volitional decision-making tasks. The results clearly favor the latter alternative. In particular, adding a time-based PM demand to performance of a challenging on-going working memory task (n-back) did not result in a measureable increase in sustained activity in any brain region, even though the on-going task was associated with widespread activity in the classic fronto-parietal cognitive control network. Moreover, the classic behavioral signature of sustained monitoring, the PM performance cost, was not present during time-based PM, even though accuracy on the PM task was high. Indeed, as discussed below, behavioral analyses revealed surprising evidence of improved on-going task performance during the period prior to when clock-checks were initiated. Thus, neither the behavioral nor the brain activity findings are consistent with a sustained monitoring account of time-based PM.

In contrast, strong evidence was observed for transient activation within aPFC, dorsolateral PFC, pre-SMA, and other fronto-parietal regions related to clock onsets. Thus, clock-related activity was significantly stronger during time-based PM than the control condition, a condition in which the clock onset was exogenously (rather than endogenously) determined (given that response instructions were provided with each clock presentation in the control condition; see Figure 1) and provided random clock times that were uninformative to task-demands Most importantly, activity was increased in these brain regions during time-based clock-checks not only following clock-onset but also in advance of it, suggesting that it was related to the volitional decision to engage in clock monitoring. Admittedly, we cannot resolve that whether such activity merely reflects a preparatory motor response, however, on the bases of our previous PM research, we believe that the patterns of activation are directly related to volitional clock checking [12], [13]).

In addition to the unique neural signature of time-based PM transient monitoring, that has not been previously observed during event-based PM, we also found that time-based PM can be distinguished from event-based PM in terms of the type of transient activation patterns observed in aPFC. Specifically, in event-based PM we have previously observed transient aPFC activity associated with responses to the PM targets [12], [13]. However, in the current time-based PM study, we found that transient activations in aPFC associated with PM responses were rather weak, at least in relationship to that observed for clock-checking behavior. This finding is consistent with the hypothesis that proximal clock-checks gradually pre-activate the PM-target response, thus requiring less additional activation to actually execute it at the correct time. Thus, taken together, the results suggest that a transient, rather than sustained monitoring process occurs uniquely during time-based PM, and that this monitoring process is indexed by subsequent clock-checking behavior.

The findings of the current study provide a new understanding of the neural mechanisms of PM, particularly the relationship between time-based PM and event-based PM. Prior work has suggested that event-based PM is frequently associated with sustained monitoring processes occurring prominently in aPFC and other frontoparietal regions [40], [13], [41]. In contrast, our multi-process account postulates that sustained monitoring is only one of multiple routes by which successful PM performance can be achieved [6], [22], [42]. In recent work, we provided evidence that sustained monitoring activity in aPFC and frontoparietal regions only occurs under certain event-based PM conditions, such as when the PM events are nonfocal to the on-going task [12]. In contrast, we demonstrated that in focal event-based conditions successful PM performance can occur even in the absence of sustained monitoring in aPFC and frontoparietal regions. The current work extends this finding by also demonstrating successful PM performance in the absence of sustained monitoring. As such, the current results are fully consistent with, and thus provide greater support for an account of PM that suggests that performance can be supported by processes other than sustained monitoring (or sustained maintenance of the PM intention [40]).

Our findings highlight the importance of investigating time-based PM, because these tasks, which are ubiquitous in daily life, may engage unique neurocognitive mechanisms to support successful task performance. Currently, however, the cognitive neuroscience literature on time-based PM is quite sparse. The first neuroimaging study reported in the literature, conducted by Okuda and colleagues [5], also reported engagement of aPFC, consistent with the current results. However, this study utilized PET methods, which because of their sluggish temporal resolution do not enable separation of transient from sustained activation. A more recent study, conducted by Haynes and colleagues [43] utilized fMRI along with multivariate pattern analysis (MVPA) methods to examine patterns of activation for information related to time-based PM, specifically the time in the future that participants were required to switch to a new task. Again, consistent with our results, bilateral aPFC regions were found to contain information related to the timing of the prospective task. However, because of the many design and analyses differences between this study and ours, a direct comparison of the results is not possible. Moreover, because this prior study used short delays (15–25 seconds) and did not allow for clock-checking, it also did not enable examination of transient versus sustained monitoring processes. On the other hand, a key advantage of the MVPA approached used by these investigators was the ability to precisely and temporally quantify the amount of information present regarding the prospective task and its duration. Further neuroscience investigations of time-based PM are needed which can capitalize and extend the findings and experimental design pioneered here, potentially through MVPA methods, to address current unresolved questions.

One such unresolved question raised by the current study concerns the functional utility of transient monitoring processes that occur in relation to clock-checking activity. The behavioral performance data reveal some interesting and potentially counter-intuitive findings in this regard. Specifically, we observed that on-going task performance was enhanced (relative to the control condition) during time-windows occurring just prior to a clock-check. This is the same time-window in which anticipatory PM-selective transient activation also occurred in aPFC and the dorsal frontoparietal network. In some sense, this finding is surprising and counter-intuitive from the perspective of transient monitoring, given that monitoring processes are typically associated with costs, rather than benefits, to on-going task performance. Moreover, as the result was unexpected, we can only speculate on an interpretation here; of course, further work is required to fully resolve the underlying mechanisms. Nevertheless, this finding is consistent with a number of possibilities, including increased arousal and/or less mind-wandering associated with clock-checking (that were associated with cognitive enhancements), or that the decision to clock-check may preferentially arise during periods of successful on-going performance (i.e., when things are “under control”). Regardless, the finding does support the idea that transient monitoring processes may have functional utility, when occurring during PM tasks.

The current findings thus suggest a functional component of transient monitoring, but it will be important for future work to provide a stronger three-way link between clock-checking behavior, brain activity dynamics, and PM task success. A potential strategy for examining this issue would be to compare these variables in populations known to exhibit impairments in time-based PM (e.g., traumatic brain injury, Parkinson’s disease, schizophrenia [44]–[46]). One particularly interesting group to study is older adults. It is now well-established that older adults show declines in PM task performance, with hints that such effects are especially prominent in time-based PM tasks [8], [33]. Moreover, a number of these studies have also demonstrated reduced clock-checking behavior in older adults as a potential source of reduced PM success [2]. Thus, an important question for future research is whether a link can be drawn between this behavioral impairment and the pattern of brain activity dynamics observed in older adults in relationship to clock-checking. For example, one possibility is that in older adults, even when clock-checking occurs, activity in aPFC could be reduced. Such a pattern would be consistent with the hypothesis that older adults do not effectively utilize clock-checking as a transient monitoring process (e.g., from which to determine the proximity to the required PM target response).

The current results are also informed by prior work examining volitional control. Specifically, we observed anticipatory activity preceding the self-initiated decision to engage in clock-checking within two regions, the aPFC and pre-SMA, that are thought to be critical components of a neural circuit for voluntary actions [30]. Admittedly, we can not rule out this activation as simply reflecting an endogenous motor response. However, our findings are similar to those of a prior study examining patterns of neural activity that predicted the timing and outcome of a volitional motor decision [47]. In that study, both pre-SMA and aPFC were identified, as regions that selectively predict the timing and particular motor response associated with a volitional decision, respectively. Interestingly, these predictive patterns of activity were observed up to 10 seconds before the voluntary decision was made in aPFC, and presumably before the intention had even entered into awareness. In our study, even taking into account the sluggishness of the hemodynamic response, anticipatory responses were only observed ∼5 seconds before clock-check activity. However, these findings are not necessarily in conflict, since, in the prior work, MVPA methods were used to detect anticipatory neural signals, whereas the current study employed the standard GLM approach [47]. Indeed, inspection of the GLM effects reported look more similar to our own findings. Thus, one promising future direction would be to employ MVPA methods in studies of transient monitoring (i.e., anticipatory clock-related activity) in time-based PM, because of their potential for increased sensitivity.

Although the current findings suggest an important role of volitional control processes in time-based PM, we interpret the neural activity patterns associated with clock-checking during time-based PM to go beyond simple volitional motor decisions. In particular, during time-based PM, not only must a volitional decision to engage in clock-checking be made, but this also must occur in relationship to the upcoming PM task (i.e., determining when the target time duration has occurred). Indeed, this is why we refer to the neurocognitive process associated with clock-checking as transient monitoring. More specifically, we postulate that an important component of transient monitoring in time-based PM is not only the decision to engage in clock-checking but also the integration of clock-related information with the prospective intention (i.e., determining how soon the PM task will need to be implemented). In contrast, for simple volitional motor tasks (e.g. [47]), there is no requirement for such monitoring of the PM goal. Thus, we hypothesize that the heightened activity in aPFC and other frontoparietal regions occurring not only prior to the clock-check response, but also after it (relative to the control condition), might reflect this form of transient monitoring and integration of clock-related information. In contrast, the pre-SMA activation might be more reflective of the volitional motor plan itself. Consistent with this interpretation, in the current results we observed a dissociation between pre-SMA and aPFC, in which the preparatory and post-response activation was greater in aPFC for clock-checks (which require temporal integration) than for the PM response, while the reverse pattern was found in pre-SMA. However, it would be possible to test this account more directly by contrasting time-based PM conditions of the type studied here with simple volitional-decision making occurring within a closely matched experimental context (e.g., asking subjects to make periodic clock-check actions while also performing an on-going task, but without any additional PM task requirement).

Although speculative, we propose that, when taken together, the findings presented here point to a common functional role for aPFC in time-based and event-based PM, but one in which this region is engaged with flexible dynamics in order to meet the specific demands of the PM task context. Specifically, we hypothesize that aPFC is important for translating the PM intention (a higher-level, abstract representation of the PM task goal) into the appropriate motor action, contingent upon the appropriate time or event occurring. In focal event-based PM conditions, aPFC may only need to be engaged transiently to activate the PM intention when this can be triggered by a highly salient cue (the PM target). In contrast, under *nonfocal* event-based conditions, aPFC may need to be engaged in a sustained fashion to tonically pre-activate the PM intention when the stimulus cues are expected to be of low salience, but still must be monitored to detect the target event that signals when the intention should be triggered (i.e., released as an action). Critically, time-based PM conditions may serve as something of a middle-ground between these two conditions. Pre-activation of the PM intention may also be required, as in *nonfocal* event-based conditions, because the external cues (i.e., on-going task stimuli) are of low salience. However, the monitoring process need only be transient, because it can directly lead to an intermediate action (clock-checking) that provides relevant information about when the PM intention should be triggered. Moreover, because clock onsets provide important timing information that enables anticipation and preparation for the PM target response, the activation needed for this event itself can be more gradual and subtle (i.e., since the response was primed, or pre-activated, by proximal clock-checks).

In summary, the findings presented here provide new information regarding the neural mechanisms that underlie time-based PM. In particular, the results reveal a transient monitoring process that supports the maintenance of prospective intentions through intermittent checking of clock information, and a transient post-clock check process that presumably integrates the temporal information to refine expectations regarding when the intention should be implemented. This latter process appears to reduce the aPFC resources engaged for actual implementation of the PM intention (relative to event-based PM). Importantly, this constellation of processes can be distinguished from the neural mechanisms previously characterized for event-based PM, which appears to rely on either sustained monitoring processes (in nonfocal PM) or bottom-up activation that occurs in the absence of monitoring (in focal PM). Finally, the experimental paradigm we have pioneered here may hold promise for further fMRI investigations of time-based PM to better characterize the changes in PM function that occur in different populations (e.g., older adults). Most generally, the results highlight the potential of neuroscience-based methods for the development of a more complete understanding of a prospective memory challenge – remembering when to perform a future act – that is ubiquitous in daily life.
